# Uncertainty-aware automatic TNM staging classification for [^18^F] Fluorodeoxyglucose PET-CT reports for lung cancer utilising transformer-based language models and multi-task learning

**DOI:** 10.1186/s12911-024-02814-7

**Published:** 2024-12-18

**Authors:** Stephen H. Barlow, Sugama Chicklore, Yulan He, Sebastien Ourselin, Thomas Wagner, Anna Barnes, Gary J.R. Cook

**Affiliations:** 1 School of Biomedical Engineering and Imaging Sciences, King’s College London, London, UK; 2 King’s College London and Guy’s and St. Thomas’ PET Centre, St. Thomas’ Hospital, London, UK; 3 Department of Informatics, King’s College London, London, UK; 4 Department of Computer Science, University of Warwick, Coventry, UK; 5 Alan Turing Institute, London, UK; 6 Department of Nuclear Medicine, Royal Free Hospital, London, UK; 7 King’s Technology Evaluation Centre (KiTEC), School of Biomedical Engineering & Imaging Science, King’s College London, London, UK

**Keywords:** Natural language processing, Deep learning, Electronic health records, Pretrained language models, Transformer, Medical imaging, PET-CT, Radiology

## Abstract

**Background:**

[^18^F] Fluorodeoxyglucose (FDG) PET-CT is a clinical imaging modality widely used in diagnosing and staging lung cancer. The clinical findings of PET-CT studies are contained within free text reports, which can currently only be categorised by experts manually reading them. Pre-trained transformer-based language models (PLMs) have shown success in extracting complex linguistic features from text. Accordingly, we developed a multi-task ‘TNMu’ classifier to classify the presence/absence of tumour, node, metastasis (‘TNM’) findings (as defined by The Eight Edition of TNM Staging for Lung Cancer). This is combined with an uncertainty classification task (‘u’) to account for studies with ambiguous TNM status.

**Methods:**

2498 reports were annotated by a nuclear medicine physician and split into train, validation, and test datasets. For additional evaluation an external dataset (*n* = 461 reports) was created, and annotated by two nuclear medicine physicians with agreement reached on all examples. We trained and evaluated eleven publicly available PLMs to determine which is most effective for PET-CT reports, and compared multi-task, single task and traditional machine learning approaches.

**Results:**

We find that a multi-task approach with GatorTron as PLM achieves the best performance, with an overall accuracy (all four tasks correct) of 84% and a Hamming loss of 0.05 on the internal test dataset, and 79% and 0.07 on the external test dataset. Performance on the individual TNM tasks approached expert performance with macro average F1 scores of 0.91, 0.95 and 0.90 respectively on external data. For uncertainty an F1 of 0.77 is achieved.

**Conclusions:**

Our ‘TNMu’ classifier successfully extracts TNM staging information from internal and external PET-CT reports. We concluded that multi-task approaches result in the best performance, and better computational efficiency over single task PLM approaches. We believe these models can improve PET-CT services by assisting in auditing, creating research cohorts, and developing decision support systems. Our approach to handling uncertainty represents a novel first step but has room for further refinement.

## Background

[^18^F] Fluorodeoxyglucose (FDG) positron emission tomography (PET), combined with computed tomography (CT) as PET-CT imaging, is widely used in determining malignancy in lung nodules and staging known lung cancer [1–3]. The clinical findings of PET-CT images are recorded in a free text report [4], and accordingly the information in these reports is difficult to extract at scale. They also contain specialist terminology and language unique to nuclear medicine which make it difficult for those without domain expertise to understand, potentially including referring oncologists and surgeons [5]. TNM (tumour, node, metastasis) staging [6] is the most widely used staging system in lung cancer to guide clinical management decisions [7] and determining how PET-CT findings relate to this staging system is essential for determining the correct treatment approach for a patient.

Natural language processing (NLP) provides methods which retrieve structured information from unstructured text and has been used in a variety of healthcare applications [8]. The significance of the written report in PET-CT offers opportunities to improve service using NLP techniques. NLP approaches in radiology across all modalities are broadly split between rule-based [9–12] and machine learning based methods [13–15]. More recently, pre-trained language models (PLMs) utilising the transformer architecture [16] have become the basis of many approaches [14, 15, 17–21]. Sykes et al. [20] found rule-based methods remain effective for biomedical tasks but time consuming to develop and potentially inflexible to external datasets. There has been less work focusing on PET-CT specifically, but recently the pre-train and fine-tune transfer learning paradigm using PLMs (demonstrated in [22]) has become prevalent for PET-CT NLP classification tasks such as sentence-level anatomy classification [19], classifying lymphoma Deauville scores [23], and distinguishing lung cancer reports from other cancers [24].

Multi-task learning techniques have also become prevalent in NLP literature [25, 26]. By training a model for multiple tasks parameter-efficiency can be increased [27], and overfitting potentially reduced due to shared knowledge between tasks [28]. These characteristics are beneficial in healthcare where computational resources can be scarce, and confidence in continued performance is important. In PET-CT Eyuboglu et al. [29] explored its benefits for image classification, but its use in classifying reports is currently underexplored.

There is some existing NLP work extracting lung cancer staging information from both PET-CT and CT reports. For PET-CT: Park et al. [30] extracted the presence and location of metastasis using convolutional neural networks and LSTM-based networks, and Nobel et al. [31] adapted a rule-based algorithm from [32] to extract T and N stage information. Neither provided extensive external validation on PET-CT reports. This is likely due to difficulties obtaining external data due to the additional burden of satisfying ethical and data protection requirements. Demonstrating a model’s efficacy on external data allows for greater understanding of performance pitfalls, and potentially allows other centres to use the model with confidence. Nobel et al. [31] also discussed the shortcomings of a rule-based approach to TN classification and how machine learning could help. For CT imaging, several approaches were developed for the “RR-TNM subtask of the NTCIR-17 MedNLP-SC shared task” using Japanese-language CT reports [33–36]. As part of this challenge Fukushima et al. [34] demonstrated the potential of fine-tuning PLMs for TNM staging classification. It should be noted that despite similarities PET-CT reports use different language from CT reports, as they primarily involve discussion of normal/abnormal tracer uptake, which is not relevant to CT reports. Accordingly, there is an opportunity to explore how PLMs can extract lung cancer staging information from PET-CT reports specifically.

TNM staging affects the treatment approach for lung cancer patients. For example, any ‘M’ positive finding would represent stage IV cancer (using the numeric system [37]) and is considered ‘advanced’ [38] cancer. Accordingly, this could significantly change the patient’s treatment plan if not known previously. It would therefore be useful to automatically identify the presence or absence of ’T’, ’N’ and ‘M’ findings in lung cancer PET-CT reports for staging, clinical alerts, and future research and audit. This process can currently only be performed reliably by having experts manually read reports. Related to this, uncertainty and ambiguity are factors in PET-CT reports [39–41]. We found previous NLP work in radiology either excluded such reports [21], or seemingly insisted the annotator decide either way [15, 30, 31]. It would be an advantage to capture this information rather than exclude or quantise it.

Accordingly, this study seeks to develop, create, and evaluate a transformer-based multi-task TNMu (Tumour, Node, Metastasis, uncertainty) classifier for FDG PET-CT lung cancer reports. It will then be evaluated on an external PET-CT dataset from another hospital with different reporting practices. Finally, we will compare its performance against human experts and analyse the results.

## Methods

### Clinical data

#### Data acquisition

MIMIC III [42] is the only public dataset with free text PET-CT report data. The newest records in this dataset are from 2012 and we found ∼ 1200 FDG PET-CT reports in total, of which ∼ 400 are for lung cancer. Due to the rapid development of PET-CT over the last decade, and the need for more reports for training and testing, we constructed a new dataset from King’s College London & Guy’s and St Thomas’ PET Centre (internal) with additional external test data from the Royal Free Hospital (external). The data use and collection was approved by UK Research Ethics Committee (UK IRAS 228790) as part of Guy’s Cancer Cohort (ref: 18/NW/0297) [43].

**Fig. 1:**
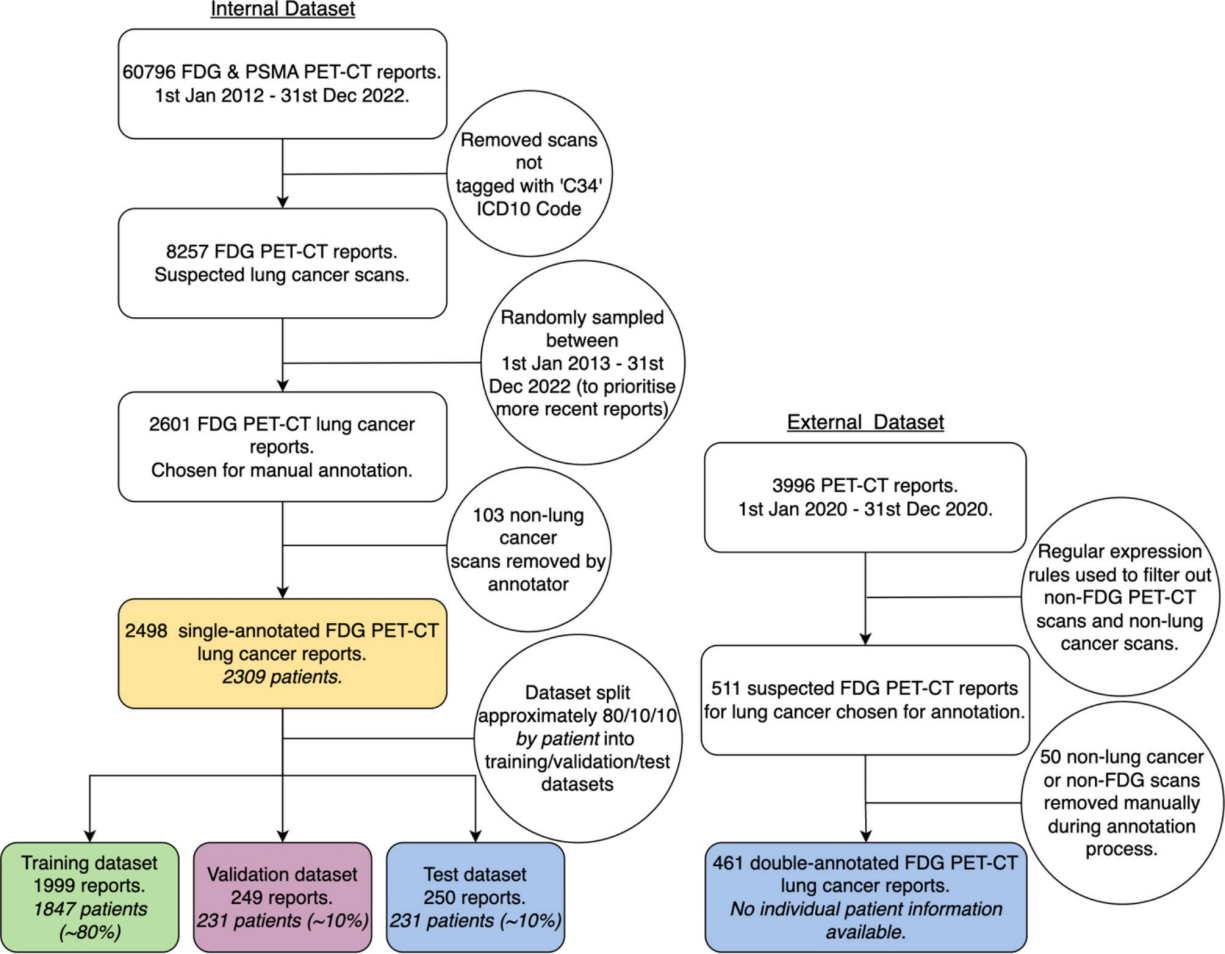
Flowcharts outlining the creation of the four datasets used in this study

We included FDG PET-CT reports with clinical indications that suspected lung cancer or investigated confirmed lung cancer. Figure 1 shows how 2601 internal reports dated between January 2013 and December 2022 were sampled from a larger corpus (*n* = 60796) for annotation. The ICD10 code ‘C34’ (malignant neoplasm of bronchus and lung) was used to identify lung cancer cases from the larger corpus. It should be noted that for the internal data source this returned both confirmed and unconfirmed lung cancer scans. As PET-CT reporting practices have developed over time we chose to represent a decade of practices in the training data of the model to encourage better generalisation to external data. During the annotation process 103 non-lung cancer reports were removed. The external data did not have ICD10 codes so 511 scans were selected for annotation using regular expression rules which searched for key terms and acronyms like ‘primary lung’ and ‘nsclc’. 461 external reports were then confirmed by the annotators as FDG PET-CT lung cancer reports and included in the final test dataset (Fig. 1).

For the internal data, anonymous patient IDs were created (and the originals permanently deleted) to create fully anonymised, stratified datasets. No other identifiable patient information was kept, and the Spacy library [44] was used to remove any names (clinicians/admin staff) and dates in the report text. The external data were fully anonymised as no identifiable patient information was provided but were also processed with the Spacy pipeline. Patient stratification was not required for the external data as they were only used for model testing.

#### Data annotation

A nuclear medicine consultant (30 years of PET experience) annotated the internal reports at the document level for the presence or absence of any finding that would represent ‘T’, ‘N’ or ‘M’-positive status according to The Eighth Edition of TNM Staging for Lung Cancer [37]. Two nuclear medicine consultants (30 and 14 years experience) annotated the external data, one being the internal annotator. We found that in some reports the original reporter would be unsure of a finding or unclear in describing it. We assert it is useful for the model to have ambiguous examples in its training and testing data to demonstrate it could work effectively on them in deployment. In our initial exploration of the data the number of uncertain/ambiguous examples varied significantly between the TNM tasks. For example, the ‘N’ findings were rarely uncertain, but uncertain ‘T’ and ‘M’ findings were more common. From this we felt creating individual ‘uncertainty’ classes for ‘T’, ‘N’ and ‘M’ would hurt performance with little benefit. To solve this, we introduce an ‘uncertainty’ (‘u’) task with the following definition:

If the annotator deems any of the TNM findings to be uncertain, indeterminate, or ambiguous then the corresponding ‘T’, ‘N’ or ‘M’ class is positive, and the ‘u’ class also becomes positive. If there is no uncertainty/ambiguity associated with the TNM findings, then the ‘u’ class is negative.

Four types of uncertainty became apparent during annotation: Reporter uncertainty (where the original reporter is uncertain of a findings significance), uncertainty from unclear language (where the original report text is unclear), uncertainty by omission (where a crucial detail is missing from the original report), and uncertainty from technical limitations (where limitations of PET-CT imaging restrict a definitive opinion). The phrase “middle lobe and left lower lobe nodules are below the resolution of PET but remain suspicious and require ongoing surveillance as a minimum” provides an example of both reporter uncertainty and technical uncertainty as the reporter is unsure of the significance of lung nodules due to technical limitations of PET-CT imaging. Linguistic clarity and omission were rarer types of uncertainty and usually came about because of unusual clinical contexts involving the whole report. Accordingly, each report was annotated with binary labels for ‘T’, ‘N’, ‘M’ and ‘u’ findings, preserving uncertainty information which would otherwise be lost via exclusion or quantisation, while allowing for consistent classification performance.

Due to the expense and time-pressures of nuclear medicine physicians we used a single annotator for the internal data, and two annotators for the external test data. If the two annotators had contradictory annotations, then these were resolved via discussion and consensus once all examples had been labelled. This allowed us to test inter-annotator agreement and create a suitable gold standard test set for final model evaluation. This annotation approach was the best use of available resources as deep learning models have been shown to get good performance from imperfect training data [29, 30, 45], but still require evaluation on gold standard data.

#### Dataset splits

The annotated internal data were split at patient level into training, validation, and test sets at a ratio of 80:10:10 (Fig. 1). Splitting datasets by patient has been used in similar studies [15, 21, 30], and serves as a simulation of external data by making sure no patient is present in more than one dataset. The external data were exclusively used for final testing to assess the model’s ability to generalise to data from another centre.

Table 1 Shows the distribution of classes for each dataset. All tasks have class imbalances with the most severe being the uncertainty task. The tumour task has a class imbalance opposite to the others with mostly positive cases. T0 (primary tumour-negative) labels are common due to PET-CT’s use in the diagnosis of lung cancer, where it can help determine if a nodule is malignant or not. Cohen’s kappa (κ) was used to test inter-annotator agreement on the external data and κ = 0.77 for ‘T’, 0.94 for ‘N’, 0.82 for ‘M’, and 0.38 for ‘u’. This represents “substantial agreement” for ‘T’, “almost perfect agreement” for ‘N’ and ‘M’, and “fair agreement” for ‘u’ according to [46].

**Table 1:** Class label distribution for the four datasets used in this study in the format ‘0/1’ where ‘0’ represents the number of negative examples for that class and ‘1’ represents the number of positive examples of that class present in the dataset

Dataset	Task Name (Absence / Presence)			
	Tumour (‘T’) (0/1)	Node (‘*N*’) (0/1)	Metastasis (‘M’) (0/1)	Uncertainty (‘u’) (0/1)
Internal Train	472 / 1527	1321 / 678	1609 / 398	1700 / 299
Internal Validation	55 / 194	160 / 89	189 / 60	213 / 36
Internal Test	54 / 196	157 / 93	194 / 56	208 / 42
External Test	89 / 372	285 / 176	336 / 125	393 / 68

### Model architecture and training

#### Model input

**Fig. 2:**
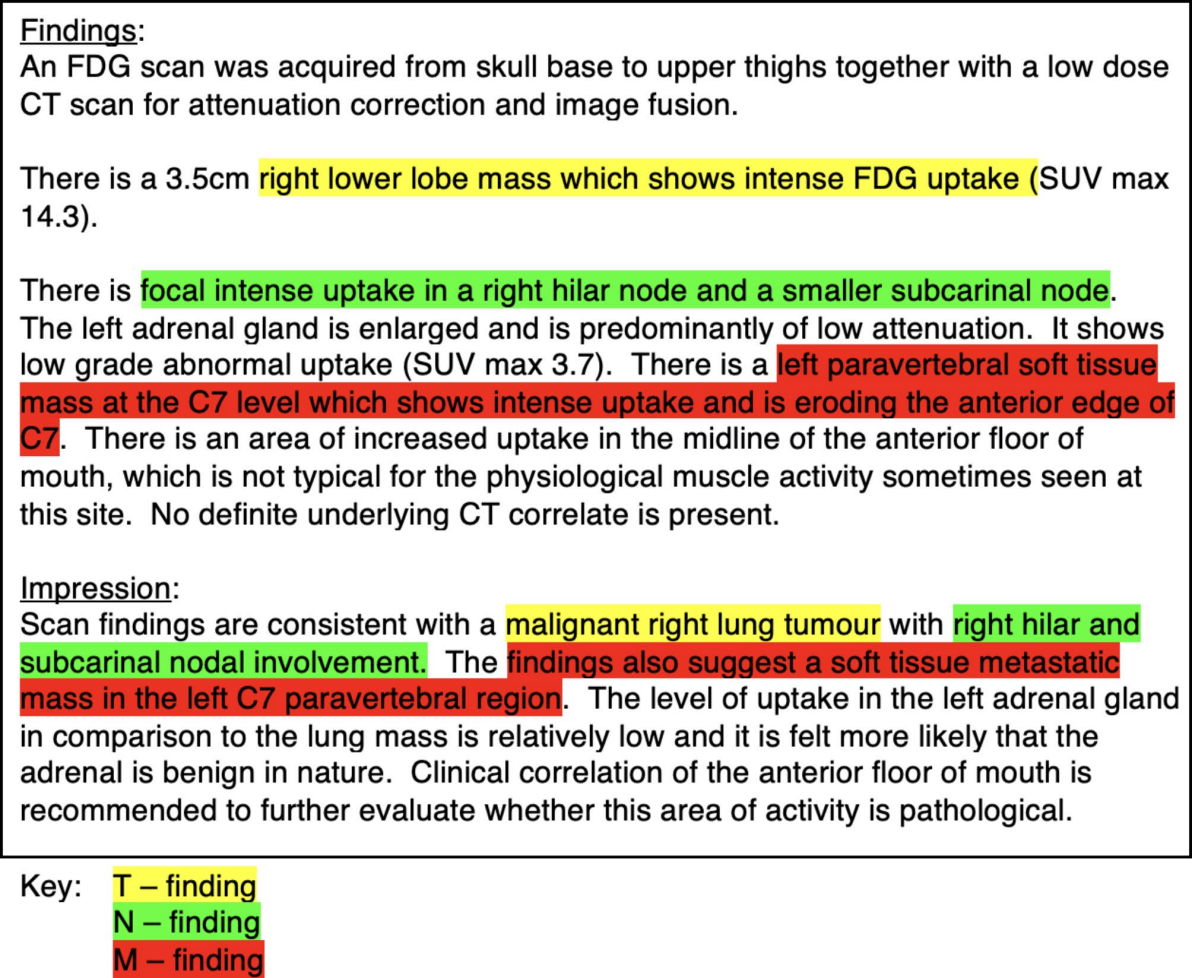
Example FDG PET-CT report. Phrases that would correspond to positive T, N or M findings are highlighted in the appropriate colour. This example would be labelled T1 N1 M1 u0 at the document level, as the original reporter has clearly noted positive Tumour, Node, and Metastasis with no ambiguity or uncertainty

PET-CT reports are usually split into ‘Findings’ and ‘Impression’ sections [39]. The exact section names can vary but keep similar semantics [47]. The ‘Impression’ section serves as the conclusion of a report as reporting guidelines [1] recommend that the clinical relevance of any findings is stated here. The ‘Findings’ section is where the observations of interest on the image are recorded, but not necessarily what they mean for the patient. Previous work has used the ‘Impression’ section alone [13, 21, 30], the ‘Findings’ section alone [19], or both [48]. For our work we chose to use the entire report as we found this offered superior performance over the ‘Impression’ or ‘Findings’ sections alone. Figure 2 shows an example FDG PET-CT report with sections highlighted that would indicate TNM findings.

Transformer based PLMs use tokenization algorithms such as WordPiece [49] or Byte Pair Encoding (BPE) [50]. We use the appropriate trained tokenizer for each PLM, truncating the start of a report if it exceeded the 512 token limit (common to all models tested) as opposed to the end. This ensures we conserve the ‘Impression’ section of a report containing the most clinically relevant information. No other text processing is performed other than that required for the tokenizer (as determined by Huggingface’s [51] implementation). We sought to use as little dataset-specific cleaning as possible as this could bias the model towards specific reporting tendencies.

#### Problem formulation and model architecture

We used the Transformers [51] and Pytorch [52] libraries to develop the models. TNM staging information can be highly contextual (Fig. 2) so a model that can incorporate contextual information is essential for good performance. PLMs like BERT [22] are pre-trained on a large corpus of text in a self-supervised fashion to gain an understanding of how language works. They can then be fine-tuned for a specific task like document classification. This fine-tuning process is a form of transfer learning [53] and is the paradigm we use in this study.

**Fig. 3:**
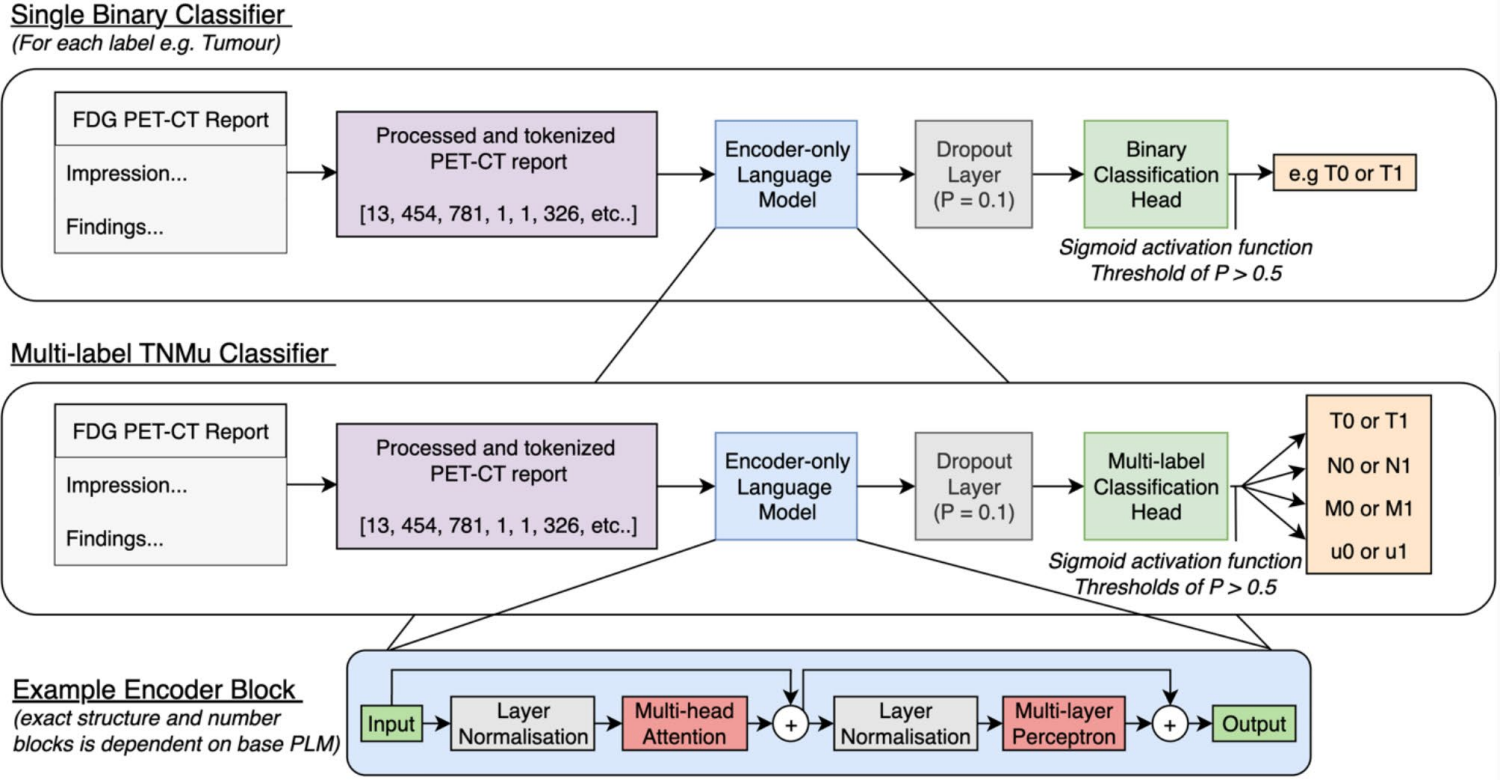
Model architecture diagrams displaying the difference between a single task approach for ‘T’ classification (which would be repeated for ‘N’, ‘M’, and ‘u’ classifications), and a multi-task approach TNMu classifier with a shared PLM encoder

We formulate the TNMu classification problem as multi-label classification, where each document is assigned four binary labels which are positive (‘1’) or negative (‘0’). PLMs offer the opportunity of using a shared encoder and utilising the benefits of multi-task learning [54, 55], allowing more appropriate features to be learnt that potentially generalise better to new data. This also makes the model more computationally efficient by reducing the number of trainable parameters required for each task. A four neuron classification head is appended to a shared PLM encoder (Fig. 3), and we compare this against training separate binary classifiers for each task with their own encoders. Figure 3 demonstrates the complete pipelines. A report is tokenized, then inputted to a PLM consisting of a series of transformer encoder blocks utilising multi-head self-attention (as detailed in [16]). A dropout [56] regularization layer is then used between the PLM and the classification head (probability set at 0.1) to prevent overfitting. The model finally outputs a number between 0 and 1 for each task and if this number is greater than 0.5, it returns a positive prediction for that task.

We treat the choice of PLM as a hyperparameter and evaluate eleven encoder-only PLMs on the validation set. PLMs encode large amounts of text data via self-supervision, and those used in this study primarily use a masked language modelling objective, where the model attempts to predict the masked word(s) for large quantities of text. Encoder-only PLMs are unable to generate text like auto-regressive and encoder-decoder models (such as GPT [25, 57] and T5 [58]), but benefit from bi-directional context in their predictions, which has proven useful for text classification tasks [22]. These PLMs are trained on corpora with different characteristics, often with a particular focus. These could broadly be described ‘general’, ‘biomedical’ and ‘clinical’. BERT [22] and RoBERTa [59] use general data sources such as Wikipedia, books, and web crawl data. BioBERT [60] and BioMegatron [61] further pre-train on biomedical literature from PubMed. GatorTron [62], BioClinicalBERT [63] and RadBERT [64] contain actual electronic health records (such as radiology reports) in their training corpora. Generally biomedical models include general data as well as biomedical data, and clinical models include general, biomedical, and clinical data.

#### Training

Hyperparameters were selected by experimenting on the internal validation set. Table 2 shows all the combinations tested, with the final choices highlighted. The final choices were determined by the best classification performance. The learning rate was set at 1e-5 using an Adam optimizer with weight decay [65]. This was decayed linearly to zero over a total training time of five epochs with the first 10% of steps used as a warmup period. The loss function used was the binary cross entropy over the four tasks. For each configuration three models were fine-tuned with different random seeds allowing the reporting of the mean and standard deviation of these runs. The batch size was set at 8 as this was the largest possible for the larger models on an Nvidia RTX 3090 GPU. These hyperparameters offered consistently good, representative performance for each PLM when tested over three random seeds.

**Table 2:** This table shows the hyperparameter combinations we tested (in all permutations). The chosen values are in underlined bold. These were determined by best classification performance

Hyperparameter	Values tested (best performing in underlined bold)					
Learning rate	9e-6	**1e-5**	2e-5	3e-5	4e-5	5e-5
Epochs	2	3	4	**5**	6	7
Batch size	1	2	4	**8**	-	-
Dropout probability	0	**0.1**	0.2	0.3	0.4	0.5

### Evaluation approach

To evaluate the models, we use metrics that characterise both the overall performance of the classifier, and individual task performance. For overall performance accuracy (ACC_TNMu_) is used to gauge how many reports are classified completely correctly over all four tasks, and we also use an accuracy score which excludes ‘u’ to focus on the clinical tasks (ACC_TNM_). Hamming loss (HL_TNMu_) is also utilised as it is more lenient, and evaluates the model by penalising individual incorrect classifications, as opposed to an incorrect set of classifications, with a lower score being better.

All four tasks demonstrated class imbalance to varying degrees which needs to be considered in evaluation. Macro average F1 score is used to marry the concerns of precision and recall and has the benefit of treating classes equally regardless of imbalance. F1_TNMu_ serves as the mean of the F1 scores for each task.

To evaluate multi-task learning we compare against the single-task models and a baseline, non-deep learning model which uses TF-IDF (term frequency-inverse document frequency) [66] encodings and a logistic regression [67] classifier for each task (implemented using Scikit-learn [68]). A ‘TNM only’ multi-task model is also trained to test if the inclusion of ‘u’ training labels degrades performance.

Finally, we take the best performing multitask model (determined using the average performance across all stated metrics over both test sets) and compare against the two annotators of the external data and an ensemble of the best single task models for each task. This ensemble was created by selecting the single task classifiers with the best macro average F1 scores (averaged over both test sets) for ‘T’, ‘N’, ‘M’, and ‘u’. Receiver operator characteristic (ROC), precision-recall curves (PRC), and confusion matrices are used to compare the final model’s performance on each task individually.

## Results

Table 3 shows how the different PLMs performed on the validation set. GatorTron significantly outperforms all the other models tested by all metrics and was accordingly chosen as the base model for the TNMu models. The other interesting finding is that the smaller models (∼ 110 million parameters) struggled to classify the ‘u’ task. The only models to achieve a macro average F1 score over 0.70 for ‘u’ had at least 340 million parameters.

**Table 3:** Comparison of PLMs on the internal validation set. Each model was fine-tuned three times with different random seeds for 5 epochs. The results show the mean and standard deviation for each metric of those training runs. The training corpora focus gives an idea of the corpus the PLM was pre-trained on. Bold text indicates the best result for that metric. All F1 scores are macro averaged

PLM	Training Corpora Focus	Parameters	ACC_TNMu_ ↑	HL_TNMu_ ↓	F1_T_ ↑	F1_N_ ↑	F1_M_ ↑	F1_u_ ↑
BERT (Base) [22]	General	110 m	0.63 ± 0.03	0.13 ± 0.01	0.78 ± 0.04	0.91 ± 0.02	0.80 ± 0.02	0.46 ± 0.00
BERT (Large) [22]	General	340 m	0.67 ± 0.02	0.12 ± 0.01	0.83 ± 0.03	0.91 ± 0.01	0.81 ± 0.01	0.53 ± 0.08
RoBERTa (Base) [59]	General	125 m	0.69 ± 0.03	0.10 ± 0.01	0.83 ± 0.02	0.93 ± 0.01	0.84 ± 0.01	0.56 ± 0.08
RoBERTa (Large) [59]	General	355 m	0.77 ± 0.01	0.08 ± 0.01	0.91 ± 0.01	0.93 ± 0.01	0.87 ± 0.02	0.74 ± 0.01
BioBERT (Base) [60]	Biomedical	110 m	0.70 ± 0.01	0.10 ± 0.00	0.85 ± 0.01	0.92 ± 0.01	0.85 ± 0.01	0.50 ± 0.02
BioBERT (Large) [60]	Biomedical	340 m	0.75 ± 0.02	0.09 ± 0.01	0.91 ± 0.02	0.93 ± 0.01	0.86 ± 0.02	0.70 ± 0.02
BioClinicalBERT [63]	Clinical	110 m	0.66 ± 0.01	0.12 ± 0.00	0.81 ± 0.03	0.90 ± 0.01	0.78 ± 0.03	0.46 ± 0.00
BioMegatron [61]	Biomedical	345 m	0.76 ± 0.02	0.08 ± 0.01	0.90 ± 0.01	0.95 ± 0.01	0.85 ± 0.01	0.73 ± 0.00
RadBERT [64]	Clinical	110 m	0.62 ± 0.01	0.13 ± 0.01	0.78 ± 0.02	0.88 ± 0.03	0.79 ± 0.03	0.46 ± 0.00
RadBERT-RoBERTa-4 m	Clinical	125 m	0.71 ± 0.01	0.10 ± 0.01	0.88 ± 0.02	0.93 ± 0.01	0.84 ± 0.01	0.59 ± 0.02
GatorTron (Base) [62]	Clinical	345 m	**0.84 ± 0.01**	**0.06 ± 0.01**	**0.96 ± 0.01**	**0.96 ± 0.00**	**0.90 ± 0.01**	**0.81 ± 0.01**

Table 4 Compares multi-task and single task approaches on the internal and external test dataset over three different training runs. Results of a TF-IDF logistic regression model are also shown to serve as a baseline. For the multi-task approaches we train models including uncertainty labels (‘TNMu’) and models without (‘TNM only’) to test if including these labels degrades performance. Unsurprisingly the PLM pipelines dramatically outperform the traditional machine learning baseline. For the PLM pipelines similar performance is observed on the TNM tasks, but a multi-task approach offers significant improvements in ‘u’ task performance and generalisation, which in turn improves the overall accuracy and hamming loss metrics. The TNM only pipeline shows no significant difference in performance, justifying the use of uncertainty labels. The PLMs generalise well to external data, but a modest drop in performance remains. This is most pronounced on the ‘u’ task.

**Table 4:** A comparison of machine learning pipelines including two multi-task approaches using a shared GatorTron PLM encoder (one including and one excluding uncertainty labels in training), an ensemble of finetuned binary classifiers using GatorTron, and a traditional machine learning model using TF-IDF encodings and individual logistic regression classifiers for each binary task. Each approach was trained three times with different random seeds with the mean result and standard deviation reported. For the single task ensembles we calculate the ‘TNMu’ and ‘TNM’ metrics using the models trained from that random seed. Bold values represent the best performing pipeline for that metric on each test dataset. All F1 scores are macro averaged

Dataset	Pipeline	ACC_TNMu_ ↑	ACC_TNM_ ↑	HL_TNMu_ ↓	F1_TNMu_ ↑	F1_T_ ↑	F1_N_ ↑	F1_M_ ↑	F1_u_ ↑
***Internal Test***	Multi-task (TNMu)	**0.84 ± 0.01**	**0.86 ± 0.00**	**0.05 ± 0.00**	**0.92 ± 0.00**	0.93 ± 0.00	0.94 ± 0.00	**0.92 ± 0.01**	**0.87 ± 0.00**
	Multi-task (TNM only)	N/a	0.85 ± 0.01	N/a	N/a	0.94 ± 0.01	0.95 ± 0.01	0.89 ± 0.01	N/a
	Single task	0.80 ± 0.02	**0.86 ± 0.00**	0.06 ± 0.00	0.91 ± 0.01	**0.95 ± 0.00**	**0.96 ± 0.00**	0.89 ± 0.02	0.85 ± 0.02
	TF-IDF + Logistic Regression	0.50 ± 0.00	0.60 ± 0.00	0.16 ± 0.00	0.66 ± 0.00	0.69 ± 0.00	0.81 ± 0.00	0.69 ± 0.00	0.45 ± 0.00
***External Test***	Multi-task (TNMu)	**0.78 ± 0.01**	**0.83 ± 0.01**	**0.07 ± 0.00**	**0.88 ± 0.01**	**0.89 ± 0.02**	**0.95 ± 0.01**	0.89 ± 0.01	**0.77 ± 0.00**
	Multi-task (TNM only)	N/a	**0.83 ± 0.02**	N/a	N/a	0.88 ± 0.01	**0.95 ± 0.00**	**0.91 ± 0.02**	N/a
	Single task	0.73 ± 0.00	0.82 ± 0.00	0.08 ± 0.00	0.85 ± 0.01	0.88 ± 0.00	**0.95 ± 0.00**	0.90 ± 0.01	0.68 ± 0.02
	TF-IDF + Logistic Regression	0.52 ± 0.00	0.61 ± 0.00	0.16 ± 0.00	0.64 ± 0.00	0.49 ± 0.00	0.85 ± 0.00	0.76 ± 0.00	0.46 ± 0.00

The best performing individual multi-task GatorTron model was selected (from the three trained in table 4) as determined by its average performance across all metrics across both test sets. In Table 5 it is compared against an ensemble of the best performing single task models for each task, and both expert annotators on the external data. The multi-task model performs better on average than the ensemble, and approaches expert performance on the individual tasks. These errors compound on the overall metrics however, to create a gap in aggregate performance against the experts.

**Table 5:** The external test set is used to compare the best performing multi-task model, an ensemble of the four best performing single task classifiers (all determined by average performance across all metrics on both internal and external test datasets), and the two expert annotators. Bold values represent which AI model pipeline performed best. All F1 scores are macro averaged

	ACC_TNMu_ ↑	ACC_TNM_ ↑	HL_TNMu_ ↓	F1_TNMu_ ↑	F1_T_ ↑	F1_N_ ↑	F1_M_ ↑	F1_u_ ↑
Multi-task	**0.79**	**0.84**	**0.07**	**0.89**	**0.91**	**0.95**	0.90	**0.78**
Single task	0.74	**0.84**	0.08	0.87	0.89	**0.95**	**0.92**	0.70
Annotator 1	0.90	0.93	0.04	0.94	0.95	0.99	0.96	0.84
Annotator 2	0.89	0.93	0.04	0.93	0.94	0.99	0.95	0.83

As the training data were annotated by a single annotator, we test whether this manifests in annotator-bias on the external test set using Cohen’s Kappa. If the agreement scores are significantly higher with the annotator who labelled both sets (when compared with agreement to the other annotator) we could argue there is a degree of bias. Table 6 shows that the discrepancy is not uniform with the tumour and node tasks displaying minimal differences, however the metastasis and uncertainty tasks do display greater agreement with the training data annotator suggesting a degree of bias towards their judgement. The lower agreement scores for uncertainty reflect that this task had lower labelling agreement between annotators initially.

**Table 6:** Cohen’s Kappa is used to compare the best multi-task model’s inter-annotator agreement with each expert annotator (before agreement process) on the external test set

	Tumour (κ)	Node (κ)	Metastasis (κ)	Uncertainty (κ)
*Annotator 1*	**0.78**	0.88	**0.81**	**0.56**
*Annotator 2*	0.75	**0.89**	0.75	0.46

**Fig. 4:**
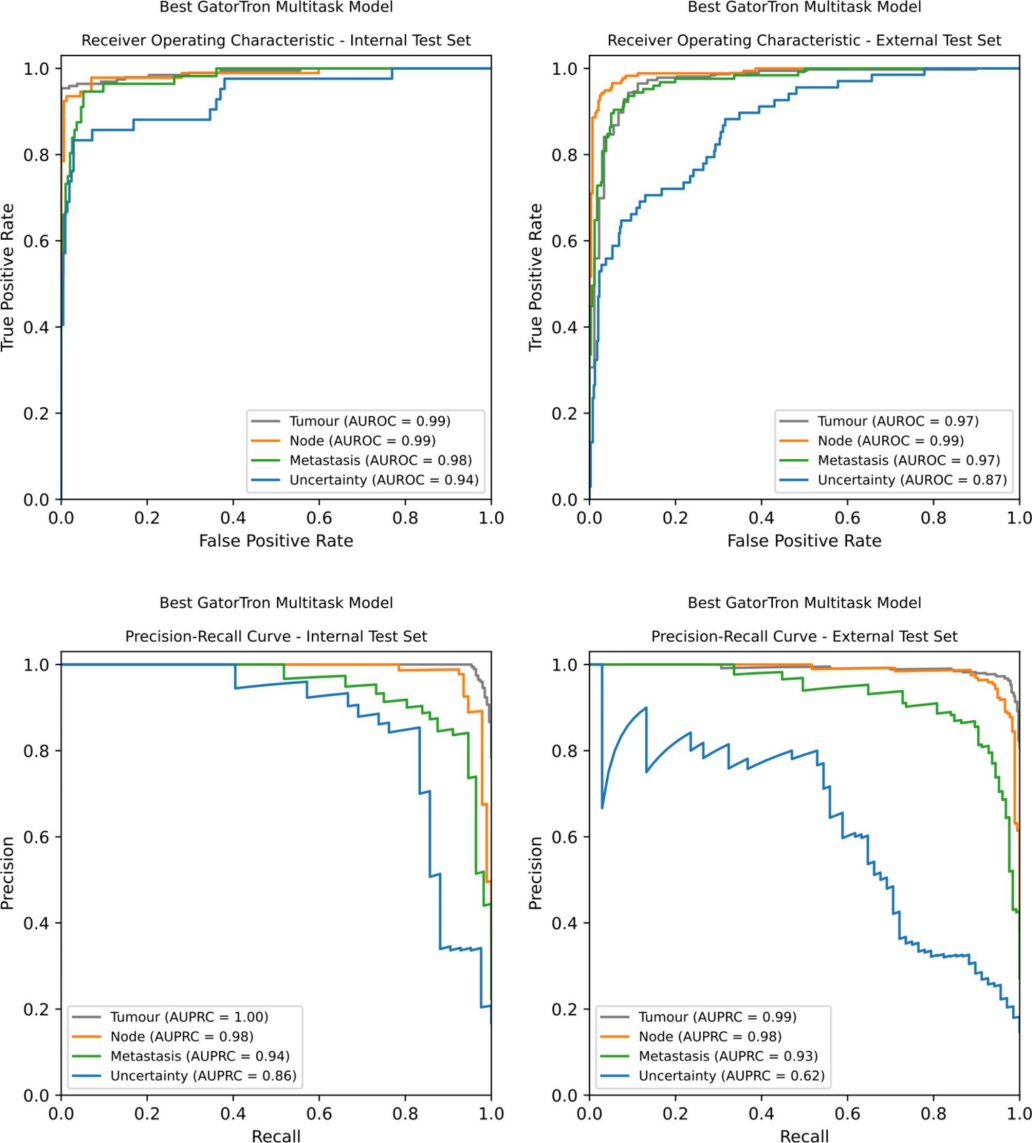
Receiver operating characteristic (ROC) and precision-recall curves for the best multi-task GatorTron model on both internal and external test sets. The ‘T’ precision-recall curve is included for completeness, but due to the class distribution being skewed towards the positive label it overstates performance and is likely not a suitable performance metric

Figure 4 shows receiver operating characteristic (ROC), precision-recall curves, and the corresponding areas under each curve for the best performing multi-task model on the internal and external test sets. The TNM tasks show similar patterns on both datasets, but the uncertainty task drops in performance on the external data. The tumour task precision-recall curve is included for completeness, but due to it having predominantly positive labels, its performance is likely overstated with precision-recall curve metrics.

**Fig. 5:**
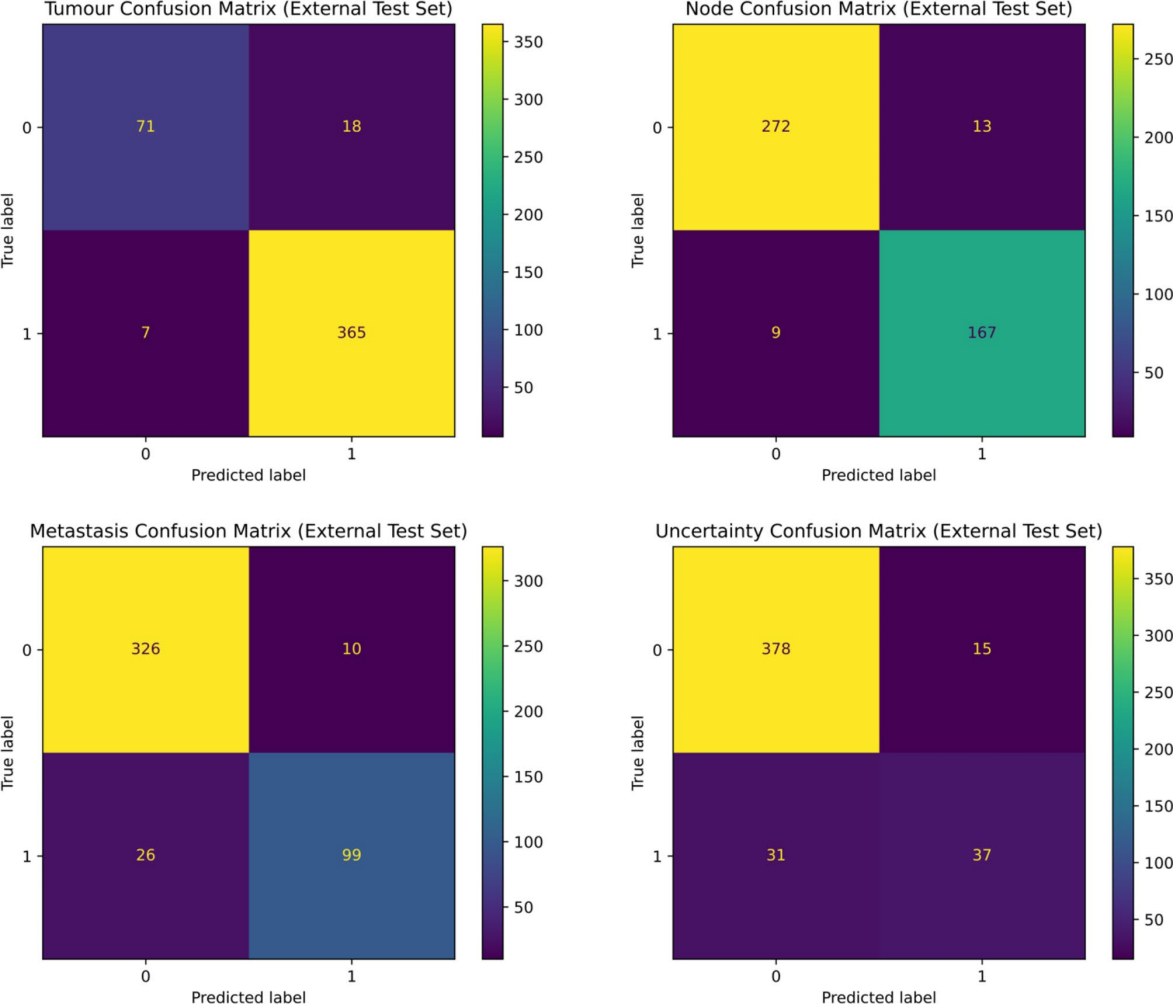
Confusion matrices for each task using the best performing multi-task GatorTron model on the external test set

Figure 5 shows confusion matrices for the model on the external dataset. The model predominantly makes correct classifications but the errors trend towards false-positives for the ‘T’ and ‘N’ tasks, and false-negatives on ‘M’ and ‘u’.

## Discussion

In this work we have developed a deep learning NLP model which classifies the TNM status of FDG PET-CT reports for lung cancer, and whether uncertainty is associated with the findings. The best performing TNMu classifier was a multi-task model utilising GatorTron [62]. This model classified 84% of internal reports, and 79% of external reports correctly across all four tasks. There was a 5% (HL_TNMu_ = 0.05) chance of it classifying an individual internal TNMu class incorrectly, and a 7% (HL_TNMu_ = 0.07) chance of incorrectly classifying an external TNMu class. We also evaluate the model against the experts’ classifications on the external data (before consensus) as humans also make errors in classifying documents. Despite not matching expert performance, the model approaches it, particularly on the clinical TNM tasks, and is capable of categorising large numbers of reports in a fraction of the time, which would be particularly useful for audit purposes, and creating research cohorts. We believe this classification performance makes the model applicable for both primary uses of PET-CT for lung cancer patients. Being able to distinguish between T-positive and T-negative assists the use case where a lung nodule is being characterised for malignancy, potentially before formal diagnosis, and the binary classifications of ‘N’ and ‘M’ status assist staging known lung cancers, where positive findings can significantly impact treatment pathways for patients going forward. A last potential use is to use these report models as a noisy labeller for PET-CT images themselves to create larger datasets for a TNM image classifier. This approach was used successfully for brain MRI in [15, 69].

The main performance difference between the two datasets concerns the ‘u’ task. With this task removed from consideration the internal accuracy over the TNM tasks becomes 86% and only drops to 84% for external data. This suggests good generalisation for the clinical tasks and is encouraging as the external reporting practices are different from those used internally. The external reporters more frequently use an “anatomic” approach for the ‘findings’ section (e.g. report findings in the head and neck first, then thorax etc… [70]), as opposed to a “priority” approach prevalent internally (where the most clinically relevant findings are stated first in order of T, N and then M [70]). This suggests transformer-based NLP models can be robust to different reporting styles for TNM classification. This change in style could also offer a reason for why the ‘u’ task generalises less well. Uncertainty is not as formally defined as the TNM tasks, so it could be more dependent on individual human judgements, which could be affected by reporting style choices. This may also explain why the inter-annotator agreement for the ‘u’ task was significantly lower than for the other tasks on the external test set. Despite this limitation it was interesting that a multi-task model was able to learn more generalisable features for uncertainty than a single task approach, and we assert capturing this information noisily is better than removing it. We found no other similar work which attempted to handle uncertainty outside of removing ambiguous reports [21], and most did not mention how uncertainty/ambiguity was handled, suggesting quantisation into the positive or negative categories. Uncertainty and ambiguity in reports is a potential concern in the wider field of radiology reporting [40, 41] and with further research a model could be developed as a teaching, or warning tool if report text is deemed to be too ambiguous. It is also worth noting that the discrepancy in performance between the ‘TNM’ and ‘u’ tasks would not have been as clear without the emphasis put on external evaluation, nor the improvements from multi-task learning. This confirms that external validation of deep learning models is useful for finer-grain analysis of model performance.

Looking at the TNM tasks individually it was observed that ‘N’ was the easiest for both the models and experts to classify across every experiment. We speculate this is likely this is due to being more self-evident than ‘T’ or ‘M’. Lymph nodes tend to be either abnormal or normal, as determined by increased uptake of the FDG tracer, and the language used in reports seems to reflect this. The ‘T’ task may depend on contextual information that is not be explicitly stated in every report, and ‘M’ findings can be associated with multiple organs, resulting in a wider range of descriptors. It is interesting to note that the experts’ classification decisions against the gold standard are also imperfect. Their individual classification performance and inter-annotator agreement statistics follow a similar pattern to the models where ‘N’ status is more successfully determined than ‘T’ and ‘M’. This suggests they are harder to ascertain from reading PET-CT reports than ‘N’ findings.

Developing the model has provided insights into important parts of the methodology. We found the choice of PLM to be the most crucial component of developing these systems. Other literature [60, 61] has shown that medical text benefits from specialised PLMs, but FDG PET-CT reports seem to benefit from further specialisation. GatorTron dramatically outperformed all the other PLMs tested, and we suspect this is due to the pre-training data. It was pre-trained on > 90 billion words of de-identified EHRs from the University of Florida [62]. We speculate its superior performance stems from being the only PLM found that contains contemporary PET-CT reports in its pre-training corpus. Tan et al. [21] also found GatorTron to be the best performing PLM in classifying CT radiology reports, but we note the performance difference was much smaller than witnessed in this study. It is also interesting that RadBERT, which is specialised for radiology tasks [64], and pre-trained on radiology reports, did not perform as well in comparison. This suggests that PET-CT reporting contains language that is distinct from other imaging modalities. A final feature of the PLMs tested was that smaller models (∼ 110 million parameters) struggled to classify the uncertainty task, whereas larger models (> 340 million parameters) were able to make a reliable distinction.

Multi-task learning was also found to provide benefits to performance and computational (and therefore also energy) efficiency, as has previously been described in other work [25–27, 54, 55]. Using a single 345 million GatorTron encoder out-performs individual classifiers for TNMu classification. This was true even when creating an ensemble from the best performing individual classifiers. This approach also reduces both the time and computation required for both training and inference, as our multi-task approach adds only three trainable parameters to the model (∼ 0.00000087%). All this suggests there is no downside to experimenting with multi-task approaches, yet many potential upsides. It is possible that reformulating other medical classification tasks as multi-label problems may provide similar benefits, although this is likely to be task dependent.

The main limitation of this study was how much expert annotated data we could accrue. Annotation is the most expensive part of the project, as experienced nuclear medicine physicians are required to make the judgements we are trying to model. Accordingly, we used a single annotator on the internal data. This means the model will potentially be biased towards that annotator’s judgement, and annotation mistakes made during that process could affect its classifications. We attempted to explore how much bias is encoded into the model by evaluating Cohen’s Kappa against the training data annotator, and the second annotator on the external data. Interestingly, there seems to be negligible bias on ‘T’ and ‘N’ tasks, but more on the ‘M and ‘u’, potentially because these tasks require more personal experience in the decision process. By evaluating the model on external gold standard data labelled by two annotators with a consensus process, and by quantifying the potential level of annotator bias in the model, we hope to mitigate most of the concerns a single annotator training dataset might create. Another limitation is that inter-annotator agreement on the ‘u’ task (κ = 0.38) is lower than would be ideal for a clinical task. This is likely due to the amount of personal experience that enters in to determining a class with no formal definition like the TNM tasks. As the ‘u’ task does not directly affect clinical decision making, and we feel there is a need to address uncertain or ambiguous reporting, this work represents a first step while conceding that more refinement is required. We also note that we did not have access to certain patient demographic information (e.g. race, gender) from either hospital. Accordingly, we cannot exclude the possibility that the model performs differently on certain demographics and cannot report detailed demographic information about the datasets used in this study. Finally, these models are specifically trained and tested on FDG PET-CT reports for confirmed or suspected lung cancer. TNM staging is defined in relation to specific cancers so performance on other cancers or other imaging modalities cannot be guaranteed.

For future work we are interested in exploring multi-modal techniques, potentially combining NLP approaches with structured data such as radiomic features, which have been shown to have good predictive value [71, 72]. Multi-modal models have been applied to text and images for CT classification tasks [73], showing promising performance, but less work has been done utilising radiomics in a multi-modal (and PET-CT) context.

## Conclusions

We created a multi-task transformer-based NLP model which successfully classifies lung cancer FDG PET-CT radiology reports for the presence or absence of tumour, node, metastasis findings and whether the report contains uncertain or ambiguous findings for these. We successfully demonstrate it performs on a dataset from another hospital with a different reporting style. We believe this has the potential to assist the creation of research cohorts, the development clinical alert systems for previously unknown findings, and to assist auditing. The uncertainty/ambiguity classification represents a novel first step, but further refinement is needed.

## Data Availability

The data presented in this study are available on request from the corresponding author. Patient report data used in this study are not publicly available for ethical reasons. Reference Python code as used to develop and evaluate models is available at https://github.com/stephenhbarlow/TNM-classification. For the purpose of open access, the author has applied a Creative Commons Attribution (CC BY) licence to any Author Accepted Manuscript version arising.
